# Developmental conditions modulate DNA methylation at the glucocorticoid receptor gene with cascading effects on expression and corticosterone levels in zebra finches

**DOI:** 10.1038/s41598-019-52203-8

**Published:** 2019-11-01

**Authors:** Blanca Jimeno, Michaela Hau, Elena Gómez-Díaz, Simon Verhulst

**Affiliations:** 1 Groningen Institute for Evolutionary Life Sciences, University of Groningen, Groningen, The Netherlands; 2 Max Planck Institute for Ornithology, Seewiesen, Germany; 3 Present Address: University of Montana, Missoula, MT United States; 4 University of Konstanz, Konstanz, Germany; 5 Instituto de Parasitología y Biomedicina “López-Neyra”, CSIC, Granada, Spain; 6 Estación Biológica de Doñana, CSIC, Sevilla, Spain

**Keywords:** Ecology, Physiology

## Abstract

Developmental conditions can impact the adult phenotype via epigenetic changes that modulate gene expression. In mammals, methylation of the glucocorticoid receptor gene *Nr3c1* has been implicated as mediator of long-term effects of developmental conditions, but this evidence is limited to humans and rodents, and few studies have simultaneously tested for associations between DNA methylation, gene expression and phenotype. Adverse environmental conditions during early life (large natal brood size) or adulthood (high foraging costs) exert multiple long-term phenotypic effects in zebra finches, and we here test for effects of these manipulations on DNA methylation and expression of the *Nr3c1* gene in blood. Having been reared in a large brood induced higher DNA methylation of the *Nr3c1* regulatory region in adulthood, and this effect persisted over years. *Nr3c1* expression was negatively correlated with methylation at 2 out of 8 CpG sites, and was lower in hard foraging conditions, despite foraging conditions having no effect on *Nr3c1* methylation at our target region. *Nr3c1* expression also correlated with glucocorticoid traits: higher expression level was associated with lower plasma baseline corticosterone concentrations and enhanced corticosterone reactivity. Our results suggest that methylation of the *Nr3c1* regulatory region can contribute to the mechanisms underlying the emergence of long-term effects of developmental conditions in birds, but in our system current adversity dominated over early life experiences with respect to receptor expression.

## Introduction

Environmental conditions experienced during development can induce phenotypic changes that last for life^1–3^. This is an intriguing finding, given that almost all body materials are replaced on a regular basis, raising the question how such effects are maintained. One way in which early-life experiences can have a persistent impact on adult phenotype is via epigenetic mechanisms, i.e. through changes in DNA that do not involve changes in DNA sequence^4–6^. These mechanisms allow the integration of intrinsic and environmental signals in the genome and can lead to activation or suppression of gene function^7^. Several mechanisms of epigenetic modulation of gene function have been described, of which DNA methylation (i.e. the addition of methyl groups to the DNA) has a prominent role in the regulation of gene expression across taxa^8–10^. In vertebrates, methylation mostly takes place in cytosines that occur before guanines (CpGs), and hypermethylation of the promoter is generally linked to repression of gene expression^11–13^. Previous results in rodents and humans show that adverse conditions during early life can induce increased DNA methylation, mediating long-term phenotypic changes^5,12,14,15^, including neurobehavioural disorders in humans (i.e. anxiety, depression or bipolar disorder, reviewed in^6^).

The glucocorticoid receptor gene (*Nr3c1)* in particular has been shown to be sensitive to early-life environmental conditions, and this effect has been attributed to epigenetic mechanisms such as DNA methylation^4,16–19^. Glucocorticoids (GCs) are steroid hormones produced by the hypothalamus-pituitary-adrenal (HPA) axis. These hormones regulate behavioral and physiological processes that are instrumental for coping with environmental change, in particular when environmental change affects energy expenditure^20^. GC actions are regulated by the expression of two intracellular GC receptors: the mineralocorticoid (MR) and the glucocorticoid (GR) receptor. The MR has a high affinity to GCs, and is therefore saturated at lower circulating concentrations than the low affinity GR. The MR is expressed in specific tissues (e.g. hypothalamus, liver, heart), while the GR is widely expressed in most tissues and organs^21^. Adverse early-life environments can increase methylation of the *Nr3c1* regulatory region in mammals, including the promoter (reviewed in^22^). Moreover, early life-induced hypermethylation of the *Nr3c1* promoter in mice has been related to reduced levels of *Nr3c1* expression and disruption of the homeostatic mechanisms that regulate the activity of the HPA axis^12,23^. Thus, early life conditions can modulate sensitivity to GCs. However, while GC-sensitivity and GC-levels are unlikely to be independent, few studies investigating methylation in the GR gene also measured GC levels, and all human studies are necessarily non-experimental. Hence conclusions on the impact of *Nr3c1* methylation patterns on HPA axis reactivity have remained speculative^22^.

Current knowledge of methylation-mediated early life effects on the phenotype is mainly restricted to humans and rodents. In birds, a few pioneering studies have investigated early life effects on genome-wide DNA methylation^24^, or DNA methylation at specific genes^25–28^. These include one study on the GR gene in relation to early life conditions^25^, while none have studied DNA methylation and gene expression simultaneously. This may partly be attributed to the fact that the promoter region of the *Nr3c1* gene in birds has not yet been characterized. A previous study in superb starlings (*Lamprotornis superbus*) predicted a putative promoter sequence based on sequence homology with the *Nr3c1* promoter in rats^25^, but empirical data on the regulatory role of this or other sequences in the upstream region of the gene in birds are lacking.

Long-term epigenetic effects generated during early development are likely to have a fundamental impact on adult phenotype. We therefore tested whether developmental adversity (being reared in large vs. small broods) is associated with DNA methylation and expression levels of the GR gene (*Nr3c1*) in zebra finches (*Taeniopygia guttata*), and how expression levels are related to corticosterone (the main avian GC) levels in the same individuals. We predicted that harsh developmental conditions (large broods) would induce higher methylation in the regulatory region of the *Nr3c1* gene, with knock-on effects on gene expression of the GR and HPA axis reactivity (i.e. corticosterone levels and dynamics). We further tested whether such effects are dependent on the adult environment (high vs. low foraging costs), as we have previously shown long-term effects of developmental conditions on adult phenotype to depend on the adult foraging environment^3,29,30^ (Table S1).

## Methods

### Birds and experimental treatments

Subjects were sampled in the context of a long-term experiment at the University of Groningen, the Netherlands^3,29^. In brief, all birds were reared by randomly mated pairs housed in cages (one pair per cage; 80 × 40 × 40 cm), in either a small (2–3 chicks) or large (5–6 chicks) brood created through cross-fostering when the chicks were a maximum of 5 days old. These brood sizes are within the range observed in the wild^31^. Chicks that grow up in large broods spend more time begging, receive less provisioning, and gain less mass relative to nestlings in small broods^32^. We therefore interpret being reared in a large brood as a harsh developmental condition. From 35–40 days of age, young birds were separated by sex (to prevent the formation of pair bonds and minimize reproductive behavior) and housed in indoor aviaries (153 × 76 × 110 cm) with up to 40 young conspecifics (including siblings and non-siblings from large and small broods) and four adults (tutors, two of each sex) to allow for sexual imprinting. Once they reached c. 120 days of age they were moved to outdoor aviaries (310 × 210 × 150 cm) and stayed there until their natural death. Foraging conditions in the aviaries were either easy or hard: four aviaries provided an easy foraging environment with low foraging costs and four provided a hard foraging environment with high foraging costs^3^. This was achieved as follows: food was offered in boxes that were suspended from the ceiling, and there were holes in the boxes through which the birds could reach the food. Underneath the holes, there was either a perch (easy foraging), or not (hard foraging), in which case the birds had to fly to the food box and back for every seed (see^33^ for details). Each aviary was stocked with only one sex, while numbers of birds reared in small and large broods were balanced^3^. The age at the start of this experiment was on average higher than 120 days, due to the time required to build up a large enough cohort before starting the experiment, but did not differ significantly among experimental treatments^3^.

This is a long-term experiment and every year some birds (age c. 120 days) were added to the treatments to maintain c. 20 birds per aviary, and to keep the flock composition balanced with respect to rearing brood size^3,29^. Thus each aviary contained birds of different ages, ranging from 0.88 to 6.53 years in the data presented in this paper (mean = 3.35 ± 0.21 years).

### Dataset

DNA methylation was measured in a selection of samples of the experimental population collected in 2014–2015^29^. Samples were selected to yield a balanced sample of all experimental groups for each sex and a representative range of (previously established) baseline corticosterone concentrations. We balanced birds by experimental groups and their corticosterone concentrations as follows: for all individuals, we calculated the residuals of the baseline corticosterone models reported earlier (Table 2 in^29^). Residuals were calculated separately for each sex and experimental treatment combination. We subsequently randomly selected one individual from each residual corticosterone quantile for each experimental group for the methylation analysis. This resulted in a selection of 32 birds (4 experimental groups × 2 sexes × 4 quantiles). Additional sampling was carried out in 2017 for gene expression analyses, and we also measured DNA methylation in these samples. In 2017 we sampled all 15 individuals still alive of the group of 32 birds whose samples were selected previously (i.e. from samples taken in 2015), plus 5 individuals not sampled previously, selected to balance treatments and sexes in the sample (see below). The average age of birds at sampling was 2.87 ± 0.24 years for the birds sampled in 2014–2015, and 4.48 ± 0.22 years for the birds sampled in 2017.

#### Corticosterone profile

The corticosterone (CORT) data used in the present paper are a subset of the data presented in^34^, where the sampling protocol is described in detail. In brief, we determined baseline CORT concentrations by finishing the sampling within 2 min after entering the aviary. Subsequently, birds were put in an opaque cloth bag for 20 min after which the stress-induced sample was collected. We then tested the adrenal′s maximum ability to down-regulate the CORT response via negative feedback by administrating a dexamethasone (CORT analogue) injection into the pectoral muscle (1,000 μg/kg^35,36^) and taking a third blood sample at minute 80 (i.e. 60 mins after the stress-induced sample). Finally, we administered an adrenocorticotropic hormone (ACTH, CORT precursor) injection (100 IU/kg^37^) to determine the maximum CORT release capacity and took the final blood sample at minute 100 after first disturbance^34^. Samples were obtained in two daily sampling rounds, one in the morning 10:00–12:00 h, and one in the afternoon 14:00–16:00 h. The time of sampling did not affect baseline CORT levels^29^.

#### DNA methylation and gene expression

DNA methylation and gene expression levels of the *Nr3c1* gene were quantified in whole blood samples (2014–2015 and 2017 for DNA methylation, 2017 for gene expression). Given that birds have nucleated erythrocytes, and these represent >99% of the blood cells, we assume that our measurements correspond to this cell type only. Previous research suggests that epigenetic biomarkers in peripheral tissues (e.g. blood) may be used to test for long-term environmental effects on DNA methylation patterns^38–40^, also for the *Nr3c1* gene^4,18,25,41,42^, which is further supported by the fact that methylation patterns in blood have often been shown to correlate with methylation patterns in other tissues such as the brain^15,26,27^, but see^43^.

DNA methylation analyses were performed on samples taken in 2014–2015 from the selected 32 individuals (see above), within up to 4 weeks of the date on which they were sampled for CORT. Samples taken in 2014–2015 and used to measure methylation were not stored in a way suitable for measurement of gene expression, and we therefore collected additional samples for both DNA methylation and gene expression from 20 individuals in 2017 (see above). We aimed to measure baseline and stress-induced CORT in these 20 samples collected in 2017, but the CORT assay failed. We therefore relied on the data collected in 2014–2015 on the same individuals to test for associations between gene expression and CORT traits, building on our finding that repeatabilities of the CORT traits were high (35–70%^29,34^).

Blood samples in 2017 were taken as described above for baseline CORT samples. Samples for DNA were stored at 2–8 °C in 500 µL 2% EDTA buffer. In the lab, this buffer was replaced by 500 µL glycerol-storage buffer (50 mM TRIS, 5 mM MgCl, 0.1 mM EDTA, 40% Glycerol) carefully mixed and snapfrozen in liquid nitrogen to be stored at −80 °C until analysed. Samples for RNA were stored in TRI Reagent, immediately snap-frozen in liquid nitrogen and stored at −80 °C until analysed. DNA extraction was done using InnuPREP Blood DNA Mini Kit (Westburg), following manufacturer’s protocol. Extracted DNA and samples for RNA extraction were further analyzed at the Estación Biológica de Doñana (Sevilla, Spain).

### Hormone analyses

The protocol for CORT analyses is described in^29^. In brief, plasma CORT concentrations were measured using an enzyme immunoassay kit (ADI-900-097, ENZO Life Sciences, Lausen, Switzerland). In 2014, intra-plate coefficient of variation (CV; M ± SE) was 9.63 ± 5.1% and inter-plate CV was 15.23 ± 3.2% (n = 10 plates). In 2015, the intra-plate CV was 11.43 ± 7.05% and inter-plate CV was 9.99 ± 2.67% (n = 16 plates). Multiple samples taken from one individual were placed in neighboring wells on the same assay plate, but in other respects samples were randomly distributed.

### DNA methylation analyses

Extracted DNA (20 µL, 20 ng/ml) was subjected to bisulphite treatment using EZ DNA Methylation-Gold kit (Zymo Research) following manufacturer’s instructions. We first designed and tested specific primers to amplify different sequences in the regulatory region of the *Nr3c1* gene (i.e. 1.8 Kb upstream of the translation start site) based on published zebra finch nucleotide gene sequences, using bisulfite primer seeker tool (Zymo Research). Selected sequences were those containing the highest number of CpG sites. We selected the following primer pair with the highest specificity: forward 5′ GTT TTT TAT TGY GGG GAT GGT GAT AGA GTT GGA GAG TG, and reverse 5′ AAA AAT AAA AAA CAA TCA AAA TCA ACA CAA CAA ACA C. The amplified fragment (350 bp) was located 370 bp upstream of the first exon of the gene, and it contained 10 CpG sites, from which methylation in 8 sites (CpG2 to CpG9) could be quantified in all the samples (Fig. S1). CpG sites were named according to distance, starting from the closest to the exon (CpG1) to the furthest (CpG10).

PCRs (25 µL) were prepared with 1.25 µL of 10x ImmoBuffer (10x, Bioline), 0.5 µL of MgCl_2_ solution (50 mM), 0.5 µL of dNTP (100 nM), 0.5 µL of each primer (20 uM), 0.25 µL of Immolase DNA Polymerase (5 u/µL, Bioline) and 0.75 µL of DNA (20 ng/ml). Amplifications were carried out as follows: initial denaturation at 95 °C for 10 min; 30 cycles of 95 °C for 30 s, annealing at 59 °C for 30 s and extension at 72 °C for 60 s, and a final extension at 72 °C for 5 min. Amplicons were visualized on 2% agarose gels to confirm expected fragment size, and sent out for sequencing (Macrogen). Raw sequences were analyzed using FinchTV (Geospiza, 2015). Chromatograms were examined visually, and we discarded samples that were shorter or with double peaks in the chromatogram. The same person (BJ) quantified the number of methylated CpG sites on each blood sample, as well as the degree of methylation per CpG site (measured as percentage), calculated by comparing the peak height of the cytosine signal with the peak height of the cytosine plus thymine signal^44^. The final sample size for methylation analyses was 43 samples of 31 individuals (out of 52 samples of 37 individuals). Thus, DNA methylation analyses was carried out twice in 12 individuals, in samples taken two years apart (2015 and 2017). The quality of some samples (N = 7) was sufficient to score whether it was methylated or not but did not allow for an accurate quantification of the percentage of methylation per CpG site. In these cases, we only included the number of CpG sites methylated. Selection and scoring of samples was done blindly with respect to bird identity, treatment and other sample related variation.

### Gene expression analyses

RNA was extracted using Tri Reagent (MRC) following manufacturer’s protocol specification. Reverse transcription was performed using Superscript III RT (Invitrogen), according to the manufacturer’s instructions. Quantitative real-time PCR (Applied Biosystems) was carried out on cDNA using previously published gene-specific primer pairs for the GR mRNA (150 bp^45^) and the house-keeping gene ß-actin^46^. Reactions (12.5 μL) were performed in duplicate and consisted of 1 μL of cDNA (1:10), 1 μL of forward and reverse primers (10 μM), and 5 μL of KAPA SYBR FAST qPCR Master Mix (Kapa Biosystems). Cycling conditions were as follows: 10 min of initial denaturation at 95 °C, 45 cycles of 15 sec denaturation at 95 °C, 30 seg annealing at 58 °C, 30 sec extension at 72 °C, and a final extension of 5 min at 72 °C. The raw cycle threshold values (Ct) were converted to copy number with a standard curve (between-plate repeatability: r = 0.95, Fig. S2), and each target gene copy number was then normalized using the house keeping copy number from the same sample. Data are reported as ratio of copy numbers of GR to house-keeping. We confirmed product-specific amplification by performing melting curves for each reaction (Fig. S3), gel electrophoresis of expected sizes, and direct sequencing of amplification products. Expression levels were obtained for 19 individuals (2017), of which CORT data (from 2015) were available for 15 individuals, while methylation (same sample) was known for 10 individuals (Table 1).

**Table 1:** Final sample sizes for the different analyses presented in this study.

	DNA methylation	Gene expression	DNA methylation vs. gene expression	Gene expression vs. GC traits
N	43 (31)	19	10	15

For the traits in which some individuals were sampled twice, number of individuals is presented between brackets. See Table S2 for a breakdown of sample sizes with respect to sex and the experimental manipulations.

### Statistics

We used General Linear Mixed Models (GLMM) to test for the effect of experimental treatments on DNA methylation. We ran analogous models for the number of CpG sites exhibiting methylation (i.e. % methylation >0), and for the average percentage of methylation (as the sum of the % methylation per site divided by the number of quantified sites, including sites with zero methylation). As independent variables we included experimental treatments (foraging costs and brood size), sex and their interactions and individual identity as random factor (DNA methylation analyses). Year of sampling was not included because it explained a negligible part of the variance (<2%). To test for the consistency of methylation patterns along the amplified fragment, we ran an additional model with percentage of methylation as dependent variable and CpG site as factor (i.e. N = 8 CpG sites/sample). To test the effect of treatments on expression levels, we ran a linear model with experimental treatments as predictors.

All statistical analyses were performed using R version 3.5.3^47^ with the functions ‘lmer’ of the R package lme4^48^ and lm of the R package nlme^49^. Logarithmic transformations were performed to normalize the CORT variables. CORT responses (i.e. increases due to restraint and ACTH, and negative feedback) were calculated as ln(stress-induced CORT)–ln(Baseline-CORT), ln(ACTH-induced CORT)–ln(CORT after dexamethasone) and ln(CORT after dexamethasone) – ln(stress-induced CORT), respectively.

Inspection of the residuals confirmed negligible deviations from a normal distribution for all models, except for the associations between methylation and expression levels, which we therefore tested using (non-parametric) Spearman rank correlations.

### Ethics

All experimental procedures were carried out under the approval of the Animal Experimentation Ethical Committee of the University of Groningen, license 5150E. Methods were carried out in accordance with these approved guidelines and regulations.

## Results

### Environmental manipulations and DNA methylation

**Figure 1:**
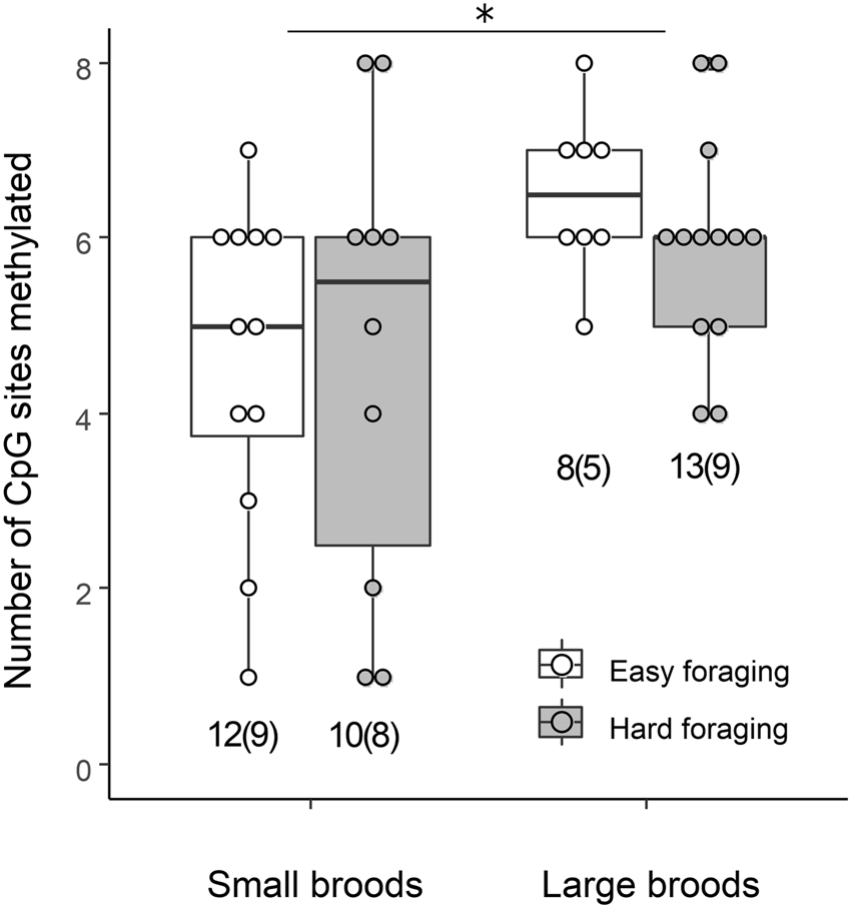
Number of methylated CpG sites in the four treatment combinations (small or large broods during development; easy or hard foraging treatment during adulthood). Numbers in bars show the number of samples per treatment, with the number of individuals in brackets, as some individuals were sampled twice (in 2015 and 2017), but note that statistical analyses controlled for individual identity. Asterisk indicates significant differences (p < 0.05).

**Figure 2:**
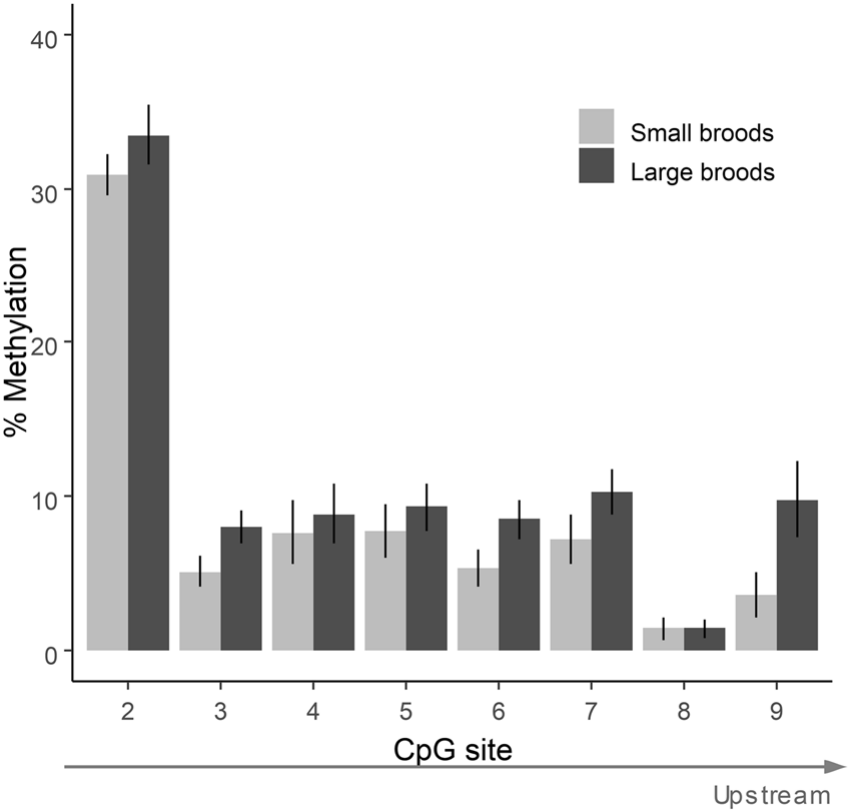
Percentage of methylation at each of the 8 CpG sites (mean ± s.e.m) quantified in birds reared in either small or large broods. Percentages were calculated over all samples, i.e. including samples/sites where % methylation was zero.

The number of methylated CpG sites (significantly: F_1,23.7_ = 7.19; p = 0.01) and the average methylation percentage per site (marginally significantly: F_1,16.8_ = 4.38; p = 0.05) were higher in individuals reared in large broods (Fig. 1), while there was no effect of foraging treatment (number of CpG sites: F_1,21.9_ = 0.09, p = 0.77; average percentage of methylation: F_1,17.1_ = 0.02, p = 0.88) or sex (number of CpG sites: F_1,21.2_ = 2.11, p = 0.16; average percentage of methylation: F_1,18.3_ = 0.23; p = 0.64, Fig. S4). The higher methylation percentage in individuals reared in large broods was consistent over CpG sites (CpG site x Brood size: F_1,250.0_ = 0.2; p = 0.66, Fig. 2). Results for 12 individuals sampled twice (in 2015 and 2017) showed consistency across years in the number of methylated CpG sites (LMM based r = 0.60, p = 0.04, Fig. S5). There was no apparent time effect on the number and methylation level of CpG sites (paired t-test, 2017 vs. 2015: t_11_ = −0.97, p = 0.35).

The percentage of methylation at CpG sites was spatially auto-correlated as evidenced by higher correlations between CpG sites located close to each other, compared to correlations between sites at a greater distance (Fig. S6).

### DNA methylation and gene expression

**Figure 3:**
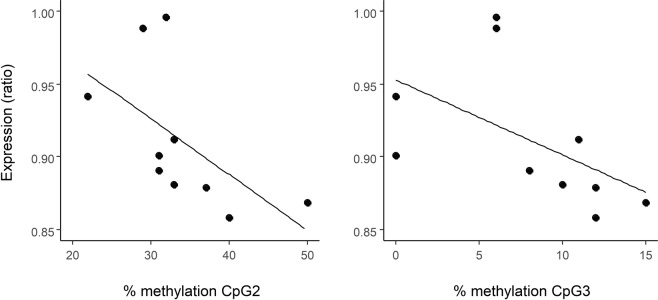
Relationship between the percentage of methylation of CpG 2 (r_s_ = −0.77, p = 0.01) and CpG 3 (r_s_ = −0.77, p = 0.01) and gene expression (reported as ratio of copy numbers of glucocorticoid receptor to house-keeping). Note that we show regression lines to guide the eye but associations were tested using a non-parametric test (Spearman-rank correlation).

Averaged over all methylation sites, *Nr3c1* expression was not significantly correlated with the number of methylated CpG sites (r_s_ = 0.07, N = 10, p = 0.85) or with average percentage of methylation (r_s_ = −0.10, N = 10, p = 0.79; Table 2). However, this association varied substantially between CpG sites and correlations were strong and significant in the expected (negative) direction at the CpG sites 2 and 3, the sites closest to the exon (Fig. 3; site 2: r_s_ = −0.77, N = 10, p = 0.01; site 3: r_s_ = −0.77, N = 10, p = 0.01). However, the spatial autocorrelation among sites (Fig. S6) argues for caution when interpreting results on single CpG sites. As there were two clusters of correlated CpG sites in our amplified region (CpG 2 and 3, and CpG 4 to 7), we also tested the relationship between DNA methylation and gene expression by summing the DNA methylation within each cluster (following^25^). These results confirmed a significant association between methylation and expression over CpG sites 2 and 3 (r_s_ = −0.78, N = 10, p = 0.01, Fig. S7), while there was no association between DNA methylation over CpG sites 3 to 7 (r_s_ = −0.22, N = 10, p = 0.54) or 4 to 7 (r_s_ = 0.02, N = 10, p = 0.95) and gene expression.

**Table 2:** Spearman rank correlation coefficients between Nr3c1 expression levels and percentage of methylation per CpG site (2 to 8), average percentage of methylation (% met), or number of CpG sites showing methylation (n°CpG).

	CpG2	CpG3	CpG4	CpG5	CpG6	CpG7	CpG8	CpG9	%met	n°CpG
**r**_**s**_	−0.77	−0.77	−0.28	−0.06	0.01	−0.13	−0.06	0.16	−0.10	0.07

### Environmental manipulations, gene expression and corticosterone traits

**Figure 4:**
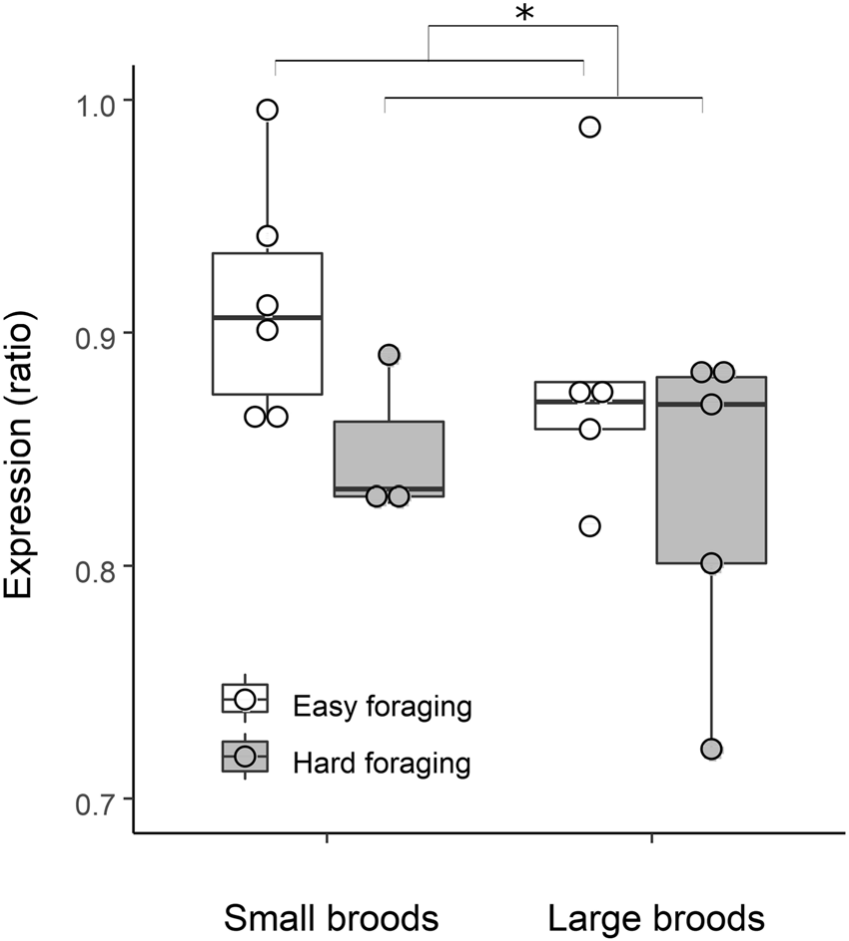
*Nr3c1* gene expression levels in the four treatment groups (small or large broods during development; easy or hard foraging treatment during adulthood). Gene expression levels are reported as ratio of copy numbers of glucocorticoid receptor to house-keeping). Asterisk indicates significant differences (p < 0.05).

**Figure 5:**
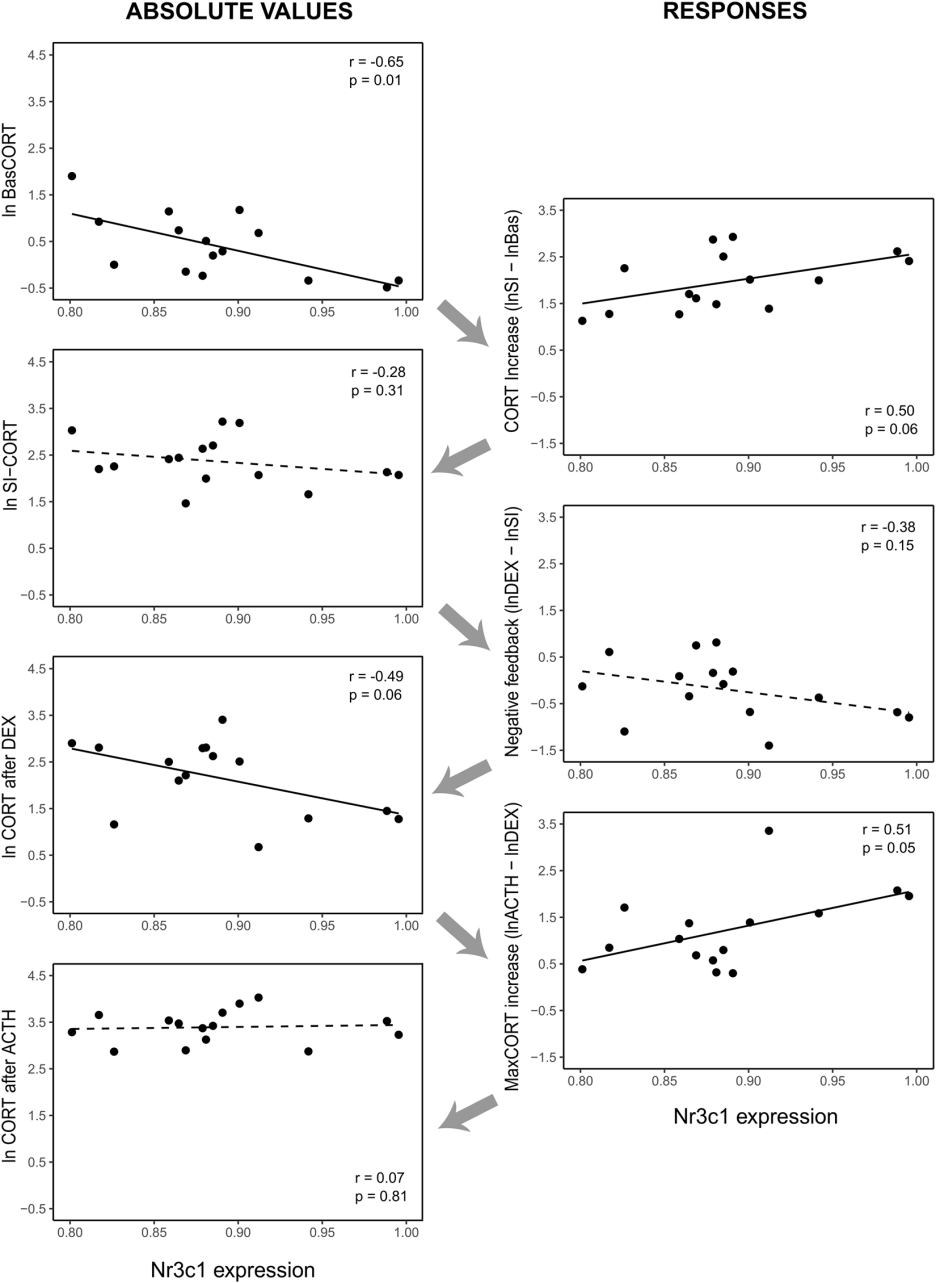
HPA regulation traits in relation to *Nr3c1* expression. Panels on the left show absolute values, while panels on the right show responses; the arrows indicate the succession of the HPA regulation steps over time. Significant (p < 0.05) or close to significance (p < 0.07) correlations are showed in continuous line, and weaker correlations are showed in dashed line. (**a**) baseline corticosterone; (**b**) corticosterone increase following restraint protocol; (**c**) stress-induced corticosterone; (**d**) feedback response due to dexamethasone injection; (**e**) corticosterone concentrations after dexamethasone injection (i.e. after feedback response); (**f**) corticosterone increase due to ACTH injection; (**g**) corticosterone concentrations after ACTH injection. Gene expression levels are reported as ratio of copy numbers of glucocorticoid receptor to house-keeping). Note Y-axis scales differ between absolute values and responses (i.e. right and left panels).

GR expression was lower in birds in the hard foraging treatment (F_1,17_ = 5.38; p = 0.03; Fig. 4), and independent of sex and brood size manipulation (Sex: F_1,15_ = 0.008, p = 0.93; Brood size: F_1,15_ = 0.89, p = 0.36). GR expression was correlated with multiple CORT traits (range r: 0.39–0.65; Fig. 5). Lower receptor expression was associated with higher baseline CORT (Fig. 5a; r = −0.65; F_1,13_ = 9.25; p = 0.01) and higher CORT increase in response to ACTH (r = 0.51; F_1,13_ = 4.55; p = 0.05; Fig. 5f). Furthermore, the correlations approached significance for lower receptor expression being associated with lower CORT increase in response to restraint (r = 0.50; F_1,13_ = 4.23; p = 0.06; Fig. 5c) and higher CORT after dexamethasone injection (r = −0.49; F_1,13_ = 4.23; p = 0.06; Fig. 5e), whereas we found no significant association between receptor expression and stress-induced CORT (r = −0.28; F_1,13_ = 1.10; p = 0.31; Fig. 5b), negative feedback (r = −0.38; F_1,13_ = 2.27; p = 0.15; Fig. 5d), and CORT after ACTH (r = 0.07; F_1,13_ = 0.06; p = 0.81; Fig. 5g).

## Discussion

Early life adversity increased methylation of the glucocorticoid receptor gene (*Nr3c1*) in zebra finches. This finding is in agreement with earlier results obtained in rodents and humans^4,6,19,23,41^, and to our best knowledge, represents the first experimental evidence on the association between early life adversity and DNA methylation in the GR gene in birds. This is also the first study reporting comprehensive results on DNA methylation, gene expression and GC traits in this animal group. DNA methylation in vertebrates is known to alter gene expression by reducing the accessibility of the promoters to the RNA polymerase transcriptional machinery^50,51^. We found that DNA methylation levels at one of the two CpG site clusters considered in the *Nr3c1* upstream region showed a reasonably strong (r_s_ = −0.77) association with gene expression, consistent with previous reports of negative associations between DNA methylation and gene expression linked to early life adversity in humans^14,15^. Our results are also in agreement with other studies finding differences between closely located CpG sites on the associations between percentage of methylation and gene expression^52^, or between the percentage of methylation and phenotypic traits (including stress reactivity^26,53^). All considered, our results suggest that DNA methylation may be one of the mechanisms mediating long-term phenotypic effects of developmental conditions, also in birds^24,25,40^. Moreover, these findings are in line with earlier studies in mammals, suggesting epigenetic programming of the GR gene to be an evolutionary conserved response to early life adversity.

Interestingly, gene expression was significantly reduced in individuals living in a hard foraging environment, while foraging conditions did not affect DNA methylation. There are multiple mechanisms that could explain this contrast and open the door towards future studies. Indeed, previous research has shown differences in GR expression independently of DNA methylation in human brain^54^. Hard foraging conditions may have modulated *Nr3c1* gene expression through methylation of other regions upstream of the gene that we did not target (see^25^), or through other epigenetic processes such as histone acetylation/deacetylation which are also important in the regulation of transcription^55^. Lastly, the foraging environment effect on gene expression may have arisen through a physiological mechanism independent of epigenetic effects. The effect of foraging treatment on *Nr3c1* gene expression in the absence of an effect on methylation gives rise to new questions regarding the relative impact of early vs. later experiences on the adult (glucocorticoid) phenotype and the mechanisms involved. These findings also suggest a sensitive phase early in life during which environmental conditions modulate methylation at the GR gene, while sensitivity is lower or zero in adulthood.

Given the brood size effect on DNA-methylation, and the association between methylation and GR expression, a negative effect of brood size on GR expression could be anticipated. However, this association did not come close to reaching significance (F_1,17_ = 1.50; p = 0.24). Nevertheless, the effects of brood size on methylation (positive and significant) and gene expression (negative and not significant) are in the expected direction, suggesting additive effects of brood size and foraging treatments on gene expression (Fig. 4). An effect of early life adversity on gene expression would be in agreement with a previous study finding altered GR expression in the brain of maternal care-deprived zebra finches^45^. The lack of significance of the brood size treatment on gene expression may be attributed to insufficient statistical power, but more work is needed to confirm (or reject) this interpretation. In any case, our findings point at current adversity dominating over early life experiences with respect to GR expression.

GCs must interact with receptors in order to exert physiological effects and when higher expression levels result in higher receptor density it can be assumed that higher expression of the *Nr3c1* gene increases GC sensitivity. It is of interest therefore that we found a negative association between baseline CORT and *Nr3c1* expression. This suggests that the optimal CORT level is lower when receptor density is high, presumably due to higher sensitivity to CORT. It also indicates that very different concentrations of baseline CORT may potentially exert the same effects on the organism, which yields an interpretation problem with respect to CORT variation that would not arise if the correlation had been positive. *Nr3c1* expression was to a variable extent associated with other GC traits: birds with lower gene expression had weaker CORT responses to ACTH, and tended to have higher CORT after dexamethasone injection, and weaker CORT responses to a standardized stressor. The GC/receptor dynamics are not static, and treatment effects on other steps of the GC regulation (e.g. GC secretion from adrenals, transport proteins, or the other intracellular receptor with higher affinity, the mineralocorticoid receptor) may also be determining the plasma GC concentrations that we measure. Genetic polymorphisms associated with several steps of GC regulation (including receptors) have also been shown to modify HPA axis reactivity at different levels^56^. Additionally, GR expression can be partly regulated by CORT concentrations, and has previously been shown to differ across tissues within the same individuals^57^. Therefore, it is possible that these relationships are partially shaped by tissue-dependent dynamics and their interplay with circulating CORT concentrations. Nevertheless, the associations between GR expression in peripheral blood and CORT phenotype that we found may help understanding the mechanistic associations between different steps of the GC regulation.

Two limitations of our study are the following. Firstly, our assumption that the DNA fragment we amplified for methylation analyses has a regulatory role in *Nr3c1* gene expression activation remains to be verified. The fragment covers a region that is located ~500 bp downstream of the putative promoter sequence described for the *Nr3c1* gene in the superb starling, the one earlier study on *Nr3c1* methylation in birds^25^. This promoter sequence was predicted by homology analysis based on the promoter sequence empirically determined in rats. An empirical demonstration that either that sequence or the one we analyzed corresponds to the actual promoter is lacking, and to what extent species differ in the location of these regulatory elements is not yet known. The fact that we found a significant association between methylation levels in this region and gene expression suggests that this region or nearby ones play a role in gene expression regulation, but this remains to be confirmed. Secondly, DNA methylation may diverge with cell type and among tissues^43,58^. The use of tissues that allow non-lethal sampling enables the collection of longitudinal data and becomes especially relevant in long-term studies, but whether different tissues show comparable epigenetic changes in response to environmental challenges or experimental manipulations remains to be investigated. Future studies are needed to examine to what extent the methylation pattern we observed in blood reflects methylations levels in tissues in which GR are most abundant (e.g. hypothalamus or liver).

An important question arising from the early development effects on *Nr3c1* methylation and cascading effects on expression and HPA axis regulation is whether such differences are adaptive in the sense that these responses enhance fitness. Most research on developmental effects and epigenetic processes has focused on detrimental medical outcomes^6,59^, but recently greater emphasis is placed on the role of epigenetic mechanisms in facilitating the adaptation of organisms to challenges later in life^5,60^. Investigating the effects of gene-specific DNA methylation and altered gene expression on survival, and whether such effects depend on developmental and adult environments, could shed new light on the functional importance and adaptive basis of epigenetic programing. Our work thus opens new perspectives towards the study of epigenetic processes in birds as pathways linking environmental conditions experienced throughout life and coping strategies, which is of fundamental and applied interest in the context of organismal adaptation in a changing world.

## Supplementary information

Supplementary Information

## Data Availability

The data generated during the current study are available from the corresponding author on reasonable request.
