# Supplementary material for: Developmental conditions modulate DNA methylation at the glucocorticoid receptor gene with cascading effects on expression and corticosterone levels in zebra finches
Source: Sci Rep. 2019 Nov 1;9:15869. doi: 10.1038/s41598-019-52203-8 (PMC6825131; doi:10.1038/s41598-019-52203-8)
Supplement: Supplementary file 1 — Supplementary Information [file 41598_2019_52203_MOESM1_ESM.pdf]

SUPPLEMENTARY INFORMATION TO:

Developmental conditions modulate DNA methylation at the glucocorticoid receptor gene with cascading effects on expression and corticosterone levels in zebra finches

Blanca Jimeno[1,2,3], Michaela Hau[2,4], Elena Gómez-Díaz[5]*& Simon Verhulst[1]*

*These authors contributed equally to this paper

1. Groningen Institute for Evolutionary Life Sciences, University of Groningen, the Netherlands.
2. Max Planck Institute for Ornithology, Seewiesen, Germany
3. Present address: University of Montana, Missoula MT, United States
4. University of Konstanz, Germany
5. Instituto de Parasitología y Biomedicina "López-Neyra", CSIC. Granada, Spain

Author for correspondence: Blanca Jimeno. University of Montana, Missoula MT, United States. Email: bjimenorev@gmail.com

Table S1. Phenotypic effects of experimental treatments (brood size manipulation during development, foraging costs manipulation during adulthood) on HPA axis regulation in our study population (data from previous studies[29,60]). The different traits correspond to the different steps of the endocrine stress response. Baseline CORT: plasma corticosterone concentrations sampled within 2 minutes after disturbance; Stress-induced CORT: plasma corticosterone concentrations sampled after 20 minutes of restraint; Feedback response: plasma corticosterone concentrations after induction of negative feedback via dexamethasone injection; ACTH-induced CORT: plasma corticosterone concentration after acute increase induced via ACTH (adrenocorticotropic hormone) injection. For details on the HPA axis characterization, see [34].

Trait	Brood size effect	Foraging costs effect	Description	Reference
Baseline CORT	Yes (females)	Yes (females)	Females from large broods had higher basCORT in hard foraging and lower basCORT in easy foraging. No effect of treatments on females from small broods, nor in males.	29
Stress-induced CORT	No	No		29
Feedback response	No (trend)	Yes	Birds in hard foraging have weaker feedback response. This difference is stronger in birds from small broods. Older birds show weaker responses, especially those from large broods in hard foraging (selective disappearance?)	61
ACTH-induced CORT	No	No		61

Table S2: Final sample sizes for the different analyses presented in this study, by sex (M=Male, F=Female), developmental treatment (S=small broods, L=large broods) and foraging treatment during adulthood (E=easy foraging, H=hard foraging. For the traits in which some individuals were sampled twice, number of individuals is presented between brackets

	**DNA methylation**							
**N**	43 (31)							
**Sex**	M				F			
	23 (16)				20 (15)			
**Brood**	S		L		S		L	
	11 (9)		12 (7)		11 (8)		9 (7)	
**Foraging**	E	H	E	H	E	H	E	H
	5(4)	6(5)	5(3)	7(4)	7(5)	4(3)	3(2)	6(5)

	**Gene expression**							
**N**	19							
**Sex**	M				F			
	10				9			
**Brood**	S		L		S		L	
	5		5		4		5	
**Foraging**	E	H	E	H	E	H	E	H
	4	1	3	2	2	2	3	2

	**DNA methylation vs. gene expression**							
**N**	10							
**Sex**	M				F			
	6				4			
**Brood**	S		L		S		L	
	2		4		3		1	
**Foraging**	E	H	E	H	E	H	E	H
	2	0	2	2	2	1	1	0

	**Gene expression vs. GC traits**							
**N**	15							
**Sex**	M				F			
	9				6			
**Brood**	S		L		S		L	
	4		5		3		3	
**Foraging**	E	H	E	H	E	H	E	H
	3	1	3	2	2	1	1	2

5'
CTGCGGCAGCGCGGGCTGGGCGCTGCCCGGGGCAGGCCGGACACCGTGTCGGAAGTTTGCCGAAGAATTT
-916
ACAGTGTTTCTCCAAATATTTATGCTCTCCGCTGGAGAAAAAAAAAATGTAGCAAATACAAACATCTGAA
-846
ACTTACGAGGTTTGTTTTCAGCGTTGGGTGTGTTTGGAGAGTAGCCGTGAACGGGTAGAGTGCTGGAAGC
-776
TGATAAAAATCCTGCTATATTTTTAGGTAGATTTTATGTATAAATTTGAGATGTATTTAAAGAATTATGC
-706
GGAGTTTACTGCGAAGTGGGTGGGTTGCATTTCGAGACTGAAGCCGTGCAAAGGTGACAAACGCTGTGTT
-636
TTGTGGTGGGGAGGTCAGGTCCTGGGGAATGCCAAACCATGGCGGTGCCGCCTCTGGGAGCTCCGGACCT
-566
GCTCTGCGAGGGGAGGGCTTTCTGGGAAAGGTGGCAAATGTCCTTTTCCCCTGCTCAAGGTGTAGTTAAA
-496
TACCAGGGAAATATACCTGGCAAAATTTGTGAACTGGGTCCTTTACCAGTTCTTACAGTTTCTTATTG**CG**
-426 -370
GGGATGGTGACAGAGCTGGAGAGTGTG**CG**CACAGATGTCA**CG**TGCTGTAGCTTGATTCTGAGTTGTATGC
-356
AGGCAGATTACTCCTTCATCCAGGTTTAATAAAGTTTTAAAATGCATAGTTCCTCCTCATTAGACAGAAA
-286
CCAAAACCTAAAAGCCCCCAAAAACAAGTGCAGTGTGTCTGAG**CG**A**CG**TGGCA**CG**TGTCAGCCCAGAG**CG**
-216
TGGTGTAC**CG**TGCCTT**CG**CTCTCACAGCAGCTGAGCCAGGTTTGCCAGG**CG**AAGGATTTAGACTTTTTGT
-146
TTTAAAACTGTTGTTAATGTGTGTTTGCTGTGCTGACTTTGACTGCTTCTCATCTTTTTATAGTTAATGG
-76 -20
TAAAGTA
-6

Figure S1. Partial sequence of the Nr3c1 regulatory region, including the amplified fragment (in grey). CpG sites within the fragment are presented in bold (first and last - 1 and 9 - CpG sites were not included in further analyses). CpG sites were named from the closest to the exon to the furthest. Negative numbers indicate the distance (in bp) from the translation start site.

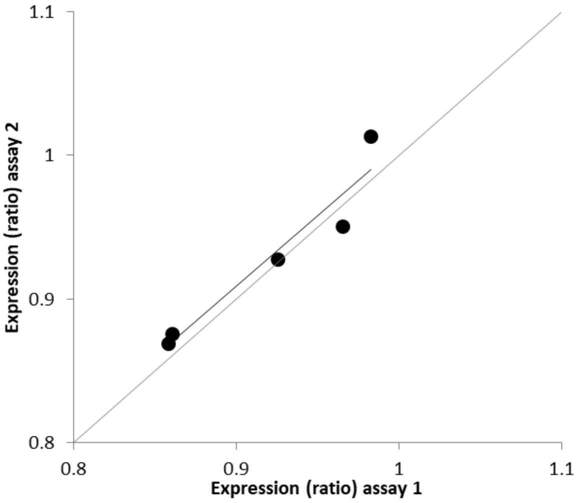

Figure S2. Expression levels (reported as ratio of copy numbers of glucocorticoid receptor to house-keeping) of 5 individuals measured on 2 different qPCR assays (r=0.95).

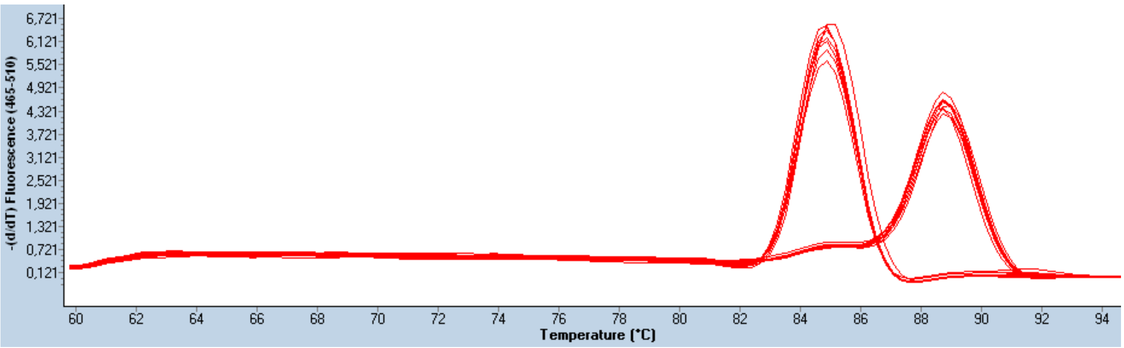

Figure S3. Melting curves of the house keeping (left) and *Nr3c1* (right) gene products in a qPCR assay.

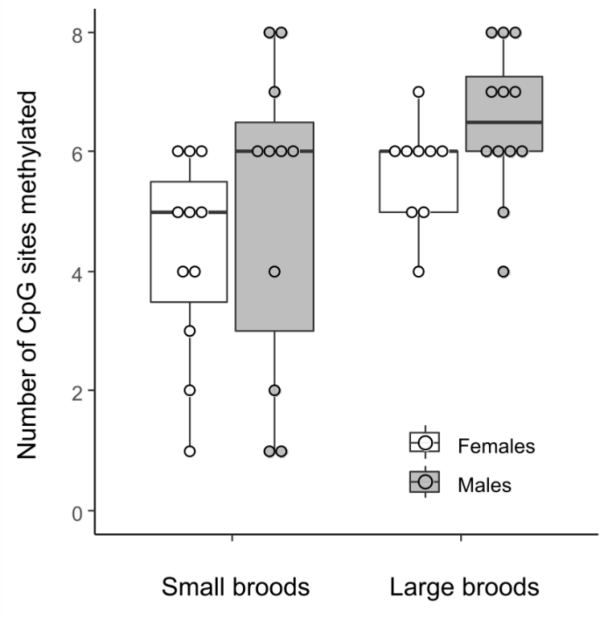

Figure S4. DNA methylation levels (number of CpG sites methylated) in males and females reared on small or large broods (developmental treatment). This figure illustrates the same pattern of higher DNA methylation in large broods occurring in males and females, but note that there were no significant differences between sexes (see results).

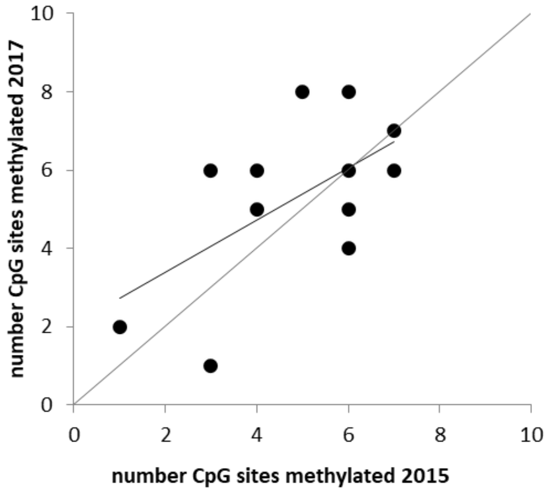

Figure S5. Correlation between DNA methylation levels of 12 individuals sampled in both 2015 and 2017 (r=0.60, continuous black line). Grey-dashed line represents y = x.

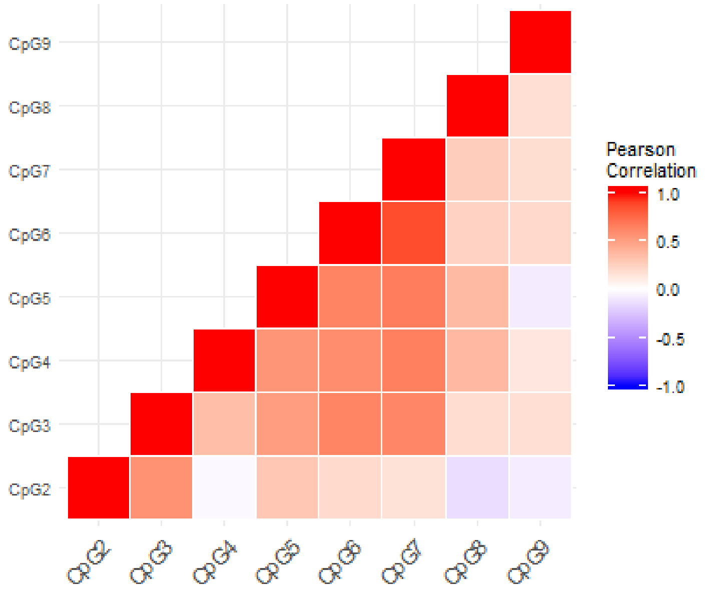

Figure S6. Pearson correlation matrix for the percentage of DNA methylation on the 8 CpG sites included in the analyses. Percentage of methylation at CpG2, the most often methylated site (and closest to the transcription region), was only significantly correlated with methylation at CpG3. Methylations at sites 3, 4, 5, 6 and 7 were significantly correlated with each other.

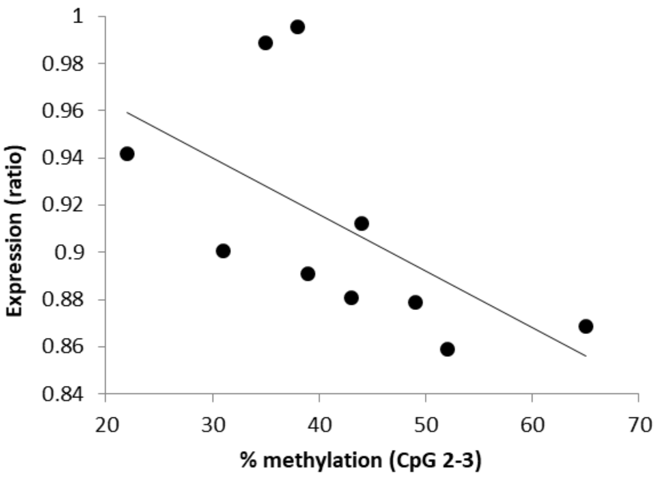

Figure S7. Relationship between the percentage of methylation in CpG 2 and CpG 3 (summed; $r_{s=}0.78$) and *Nr3c1* gene expression (reported as ratio of copy numbers of glucocorticoid receptor to house-keeping). Note that we show the regression line for convenience but associations were tested using a non-parametric test (Spearman-rank correlation; r_s= -0.78, S = 294, p=0.011)
